# Clinical efficacy and safety of antifungal drugs for the treatment of *Candida parapsilosis* infections: a systematic review and network meta-analysis

**DOI:** 10.1099/jmm.0.001434

**Published:** 2021-10-11

**Authors:** Jielin Qin, Han Yang, Zhiming Shan, Lingzhi Jiang, Qingxian Zhang

**Affiliations:** ^1^​ Department of General Medicine, Department of Respiration and Intensive Medicine, The First Affiliated Hospital of Zhengzhou University, Zhengzhou, Henan 450000, PR China; ^2^​ Department of Hepatobiliary and Pancreatic Surgery, The First Affiliated Hospital of Zhengzhou University, Zhengzhou, Henan 450000, PR China; ^3^​ Laboratory Department, Children’s Hospital Affiliated to Zhengzhou University, Henan Children’s Hospital, Zhengzhou Children’s Hospital, Zhengzhou Children Infection and Immunity Laboratory, Zhengzhou, Henan 450000, PR China; ^4^​ Department of Nephrology, The First Affiliated Hospital of Zhengzhou University, Zhengzhou, Henan 450000, PR China

**Keywords:** antifungal drugs, *Candida parapsilosis*, clinical efficacy, meta-analysis, safety

## Abstract

Antifungal drugs have already been established as an effective treatment option for *Candida parapsilosis* infections, but there is no universal consensus on the ideal target for clinical efficacy and safety of antifungal drugs for the treatment of *C. parapsilosis* infections. Few studies have directly compared the efficacies of antifungal drugs for the treatment of *C. parapsilosis* infections. We hypothesize that different antifungal drugs offer differing clinical efficacy and safety for the treatment of *C. parapsilosis* infections. We performed a comprehensive network meta-analysis on different strategies for *C. parapsilosis* infection treatment and compared the clinical efficacy and safety of antifungal drugs as interventions for *C. parapsilosis* infections. The Cochrane Database of Systematic Reviews, Medline, Embase, PubMed, Web of Science, China National Knowledge Infrastructure (CNKI), Technology of Chongqing VIP database, Wan Fang Data, and SinoMed databases were searched to identify appropriate randomized trials. Among the extracted *C. parapsilosis* cases, the survival and death rates with treatment of *C. parapsilosis* infection were compared among groups treated with different antifungal drugs. According to the evidence-network analysis, echinocandins were a better choice than other drugs for treating *C. parapsilosis* infections, and more importantly, caspofungin showed a more preferable effect for decreasing the risk of 30 day mortality. In conclusion, this study systematically evaluated the effectiveness and safety of antifungal drugs for the purpose of helping clinicians choose the most appropriate antifungal drugs. Future studies with larger samples are needed to evaluate the effects of patient factors on the clinical efficacy and safety of antifungal drugs for *C. parapsilosis* infections.

## Introduction

Invasive fungal infection has become an important factor leading to increased mortality in hospitalized patients [1, 2]. Since the 1980s, with the widespread use of corticosteroids/antibacterial drugs and the development of organ transplantation and medical life support technology, the incidence of fungal bloodstream infections has increased significantly among hospitalized patients, especially those who are immunocompromised [3]. The SENTRY Antimicrobial Surveillance Programme reported that *Candida albicans* remains the most common clinical pathogenic fungal type among hospital-acquired bloodstream infections. Meanwhile, with the increase in the incidence of fungal infections, the pathogen spectrum has changed [4]. The proportion of *Candida albicans* infections is decreasing year by year, while infections with other opportunistic pathogens such as non-*Candida albicans* including *Candida parapsilosis*, *Candida glabrata*, and *Candida tropicalis* are showing an increasing tend in prevalence and often show resistance to multiple antifungal drugs. In China, a nationwide invasive fungal infection surveillance study revealed that *Candida* species accounted for 91.3 % of yeast pathogens from invasive fungal infections, followed by *Cryptococcus neoformans* (7.0 %) and other non-candidal yeasts (1.7 %) [5]. Among these, *C. parapsilosis* as a common human skin commensal fungus is non-*albicans Candida* species [6, 7]. It does not lead to severe disease under normal immune conditions as a typical opportunistic fungus [8]. However, when patients receive broad-spectrum antibiotics or immunosuppressive treatments in the hospital, the incidence of *C. parapsilosis* infection increases significantly [7]. In recent decades, it was reported that the incidence of *C. parapsilosis* infection is rising in North America, Europe and Asia, accounting for 8–10 % of all nosocomial bloodstream infections [9, 10]. Since the first antifungal drug griseofulvin was discovered in the 1930s, antifungal drugs such as polyenes, allylamines, azoles, and echinocandins have been used to clear fungal infections. In recent years, great progress has been made in the treatment of fungal infections. Antifungal drugs including polyenes, triazoles, and echinocandins have already become effective treatment options for *C. parapsilosis* infections, even though all have some advantages and certain limitations in terms of efficacy, safety, bioavailability, and drug–drug interactions [11, 12]. Therefore, there is no universal consensus on the ideal target for clinical efficacy and safety of antifungal drugs for the treatment of *C. parapsilosis* infections [13]. Also, few studies have directly compared the efficacies of antifungal drugs for the treatment of *C. parapsilosis* infections [14].

Therefore, a comprehensive review on different strategies for *C. parapsilosis* infection treatment and a comparison of their efficacies will be meaningful and helpful for the clinical application of antifungal drugs. Accordingly, we focused on antifungal inventions in the present study, highlighting the important aspects of this therapy and performing a network meta-analysis to determine the best therapeutic choice for *C. parapsilosis* infection patients. Most importantly, the objective of this research was to provide new treatment insight extending beyond the specific therapeutic strategy itself.

## Methods

### Literature search

The following electronic bibliographic databases were searched: Cochrane Database of Systematic Reviews), Medline, Embase, PubMed, and Web of Science (science and social science citation index) as well as the Chinese databases China National Knowledge Infrastructure (CNKI), Technology of Chongqing VIP database, Wan Fang Data, and SinoMed for related studies. The search time limit was from the establishment of each database until 1 June 2020. We also manually searched collections of relevant conferences and traced the reference documents included in the research. If the information was incomplete, we contacted the authors to obtain relevant data. The search strategy included terms relating to or describing clinical trials. The search terms were ‘*Candida parapsilosis*’, ‘randomized clinical trials’ ‘randomized controlled trail’, ‘antifungal agents’, ‘treatment’, ‘itraconazole’, ‘miconazole’, ‘fluconazole’, ‘econazole’, ‘terbinafine’ and ‘terconazole’.

### Inclusion criteria

The eligible studies include randomized controlled trials (RCTs) that compared the protective effects of any antifungal drug with placebo or other antifungal drugs for *C. parapsilosis* infections. The inclusion criteria were: 1. RCT; 2. adult patients over 18 years of age, with no limit on gender or nationality; 3. interventions of antifungal drug treatment for *C. parapsilosis* infections including candidemia or invasive candidiasis; 4. outcomes included overall effectiveness, 30 day mortality, adverse drug reactions, etc.; and 5. published in English or Chinese. The exclusion criteria were: 1. non-RCT studies; 2. non-research studies such as reviews, case reports, communications, and pharmacokinetics papers; 3. duplicate publications; 4. studies with overlapping cohorts; and 5. data that could not be extracted, converted, or obtained.

### Literature quality evaluation

Two researchers (J.Q, H.Y) independently performed the literature searches and screening. After excluding studies that did not meet the inclusion criteria, including reviews, case reports and meta-analyses, the abstracts and full texts were further reviewed to determine whether they met the inclusion criteria. The extracted data included the basic information of the RCT, baseline data for patients, interventions, primary and secondary outcomes, and follow-up data. Two authors (Z.S, L.J) used the RCT Quality Evaluation Standards recommended by the Cochrane Evaluation Manual Version 5.1 to assess the methodological quality. Quality assessment included determining whether the randomization was correct, whether the allocation was blind, whether there was loss to follow-up and withdrawal, whether there was selective reporting bias, and whether there were other biases. If the information in the clinical trials was incomplete, the authors were contacted to obtain the missing data to the extent possible. The two reviewers (J.Q, H.Y) independently extracted information. The decisions recorded by the reviewers were compared and discrepancies were resolved by a third reviewer (Q.Z).

### Outcome measures

The primary outcome was the efficacy of antifungal agents (for treating invasive fungal infections). The secondary outcomes included 30 day mortality due to fungal infection, incidence of invasive candidiasis, and other probable adverse events.

### Statistical analysis

The traditional and network meta-analyses were performed with R3.5 software according to the Bayesian framework. Both direct and indirect evidence was utilized to compare the efficacy of various treatments, as described by mean differences and 95 % confidence intervals (CIs) with a significance level of 0.05. Assessment of heterogeneity among eligible studies was carried out according to Cochran’s Q-statistic and I^2^ test. The χ^2^ test was used to analyse the clinical heterogeneity and method heterogeneity of the included studies with α set at 0.1. The surface under the cumulative ranking curve (SUCRA) was adopted to rank probabilities with respect to each clinical outcome. Funnel plots were generated to investigate publication bias.

## Results

### Characteristics of included studies

**Fig. 1.**
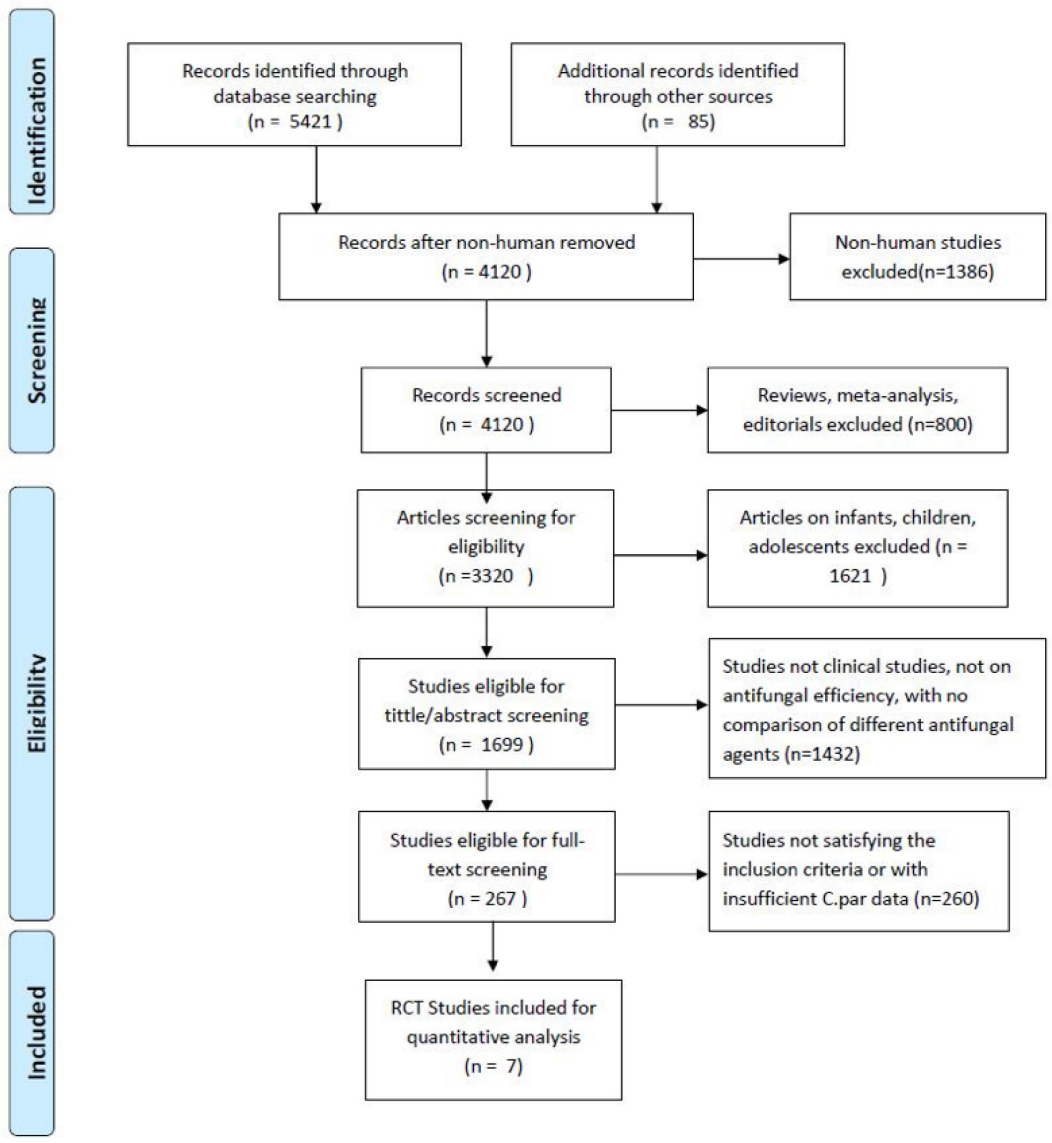
Flow diagram of the study selection process.

A total of 5506 records were identified in the primary literature search. As shown in Fig. 1, records were retrieved from the databases by searching relevant keywords, and after exclusion of case reports, editorials, comments, laboratory studies, trials involving children, and other irrelevant literature, 267 studies remained. According to the exclusion criteria, only seven out of the remaining studies were finally included in this network meta-analysis. Finally, our meta-analysis compared the clinical efficacy and safety of antifungal drugs including fluconazole (FLU), micafungin (MIC), isavuconazole (ISA), amphotericin B (AMP), caspofungin (CAS), and anidulafungin (AND) across seven studies with a total sample size of 2434 patients [15–21] (Table 1, Fig. 1).

**Table 1.** Characteristics of included randomized controlled trials studies

Study (First author)	Reported date	Study location	Total sample size	Mean age (range), years	Sex/male (%)	Type of infection	Interventions
Rex *et al*. [15]	1994	America	206	59 (na)	105 (56.0)	candidemia	FLU/AMP
Mora-Duate *et al*. [16]	2002	America	224	56 (18–84)	125 (55.8)	invasive candidiasis	CAS/AMP
Colombo *et al*. [17]	2003	America/Europe/Asia	210	52.7 (18–97)	120 (57.1)	invasive candidiasis	CAS/AMP
Pappas *et al*. [18]	2007	America/Europe/Asia	578	55.8 (24–92)	336 (58.1)	candidemia or invasive candidiasis	MIC/CAS
Reboli *et al*. [19]	2007	America	245	58.1 (24–91)	125 (51.0)	invasive candidiasis	AND/FLU
Kuse *et al*. [20]	2007	America/Europe/Asia/Africa	531	55.3 (18–84)	325 (61.2)	candidemia or invasive candidiasis	MIC/AMP
Kullberg *et al*. [21]	2019	Europe	440	57.9 (na)	269 (61.1)	candidemia or invasive candidiasis	CAS/ISA

FLU, Fluconazole; AMP, Amphotericin B; VOR, Voriconazole; CAS, Caspofungin; AND, Anidulafungin; MIC, micafungin; na, data not available.

### Risk of bias in the evidence base

**Fig. 2.**
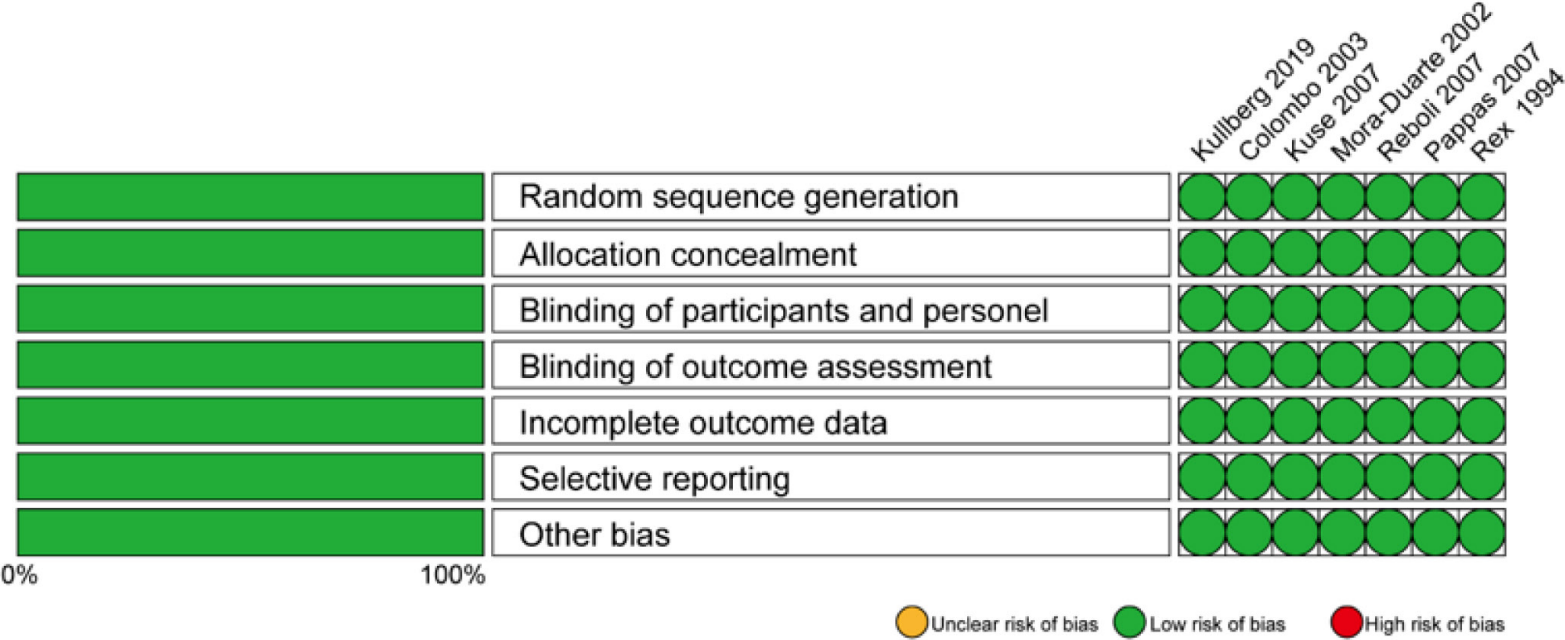
Risk of bias for all individual studies included in the analysis. The risk of bias graph shows the reviewers’ assessment of the risk of bias shown as percentages for all included studies.

All included studies were RCTs, and the quality evaluation was conducted using the criteria of the Cochrane Collaboration tool for assessing the risk of bias in RCTs (Fig. 2). All seven included RCTs satisfied the required items, including random sequence generation, allocation concealment, blinding of the study participants and personnel, and blinding of outcome assessments.

### Evaluation of antifungal treatment

**Fig. 3.**
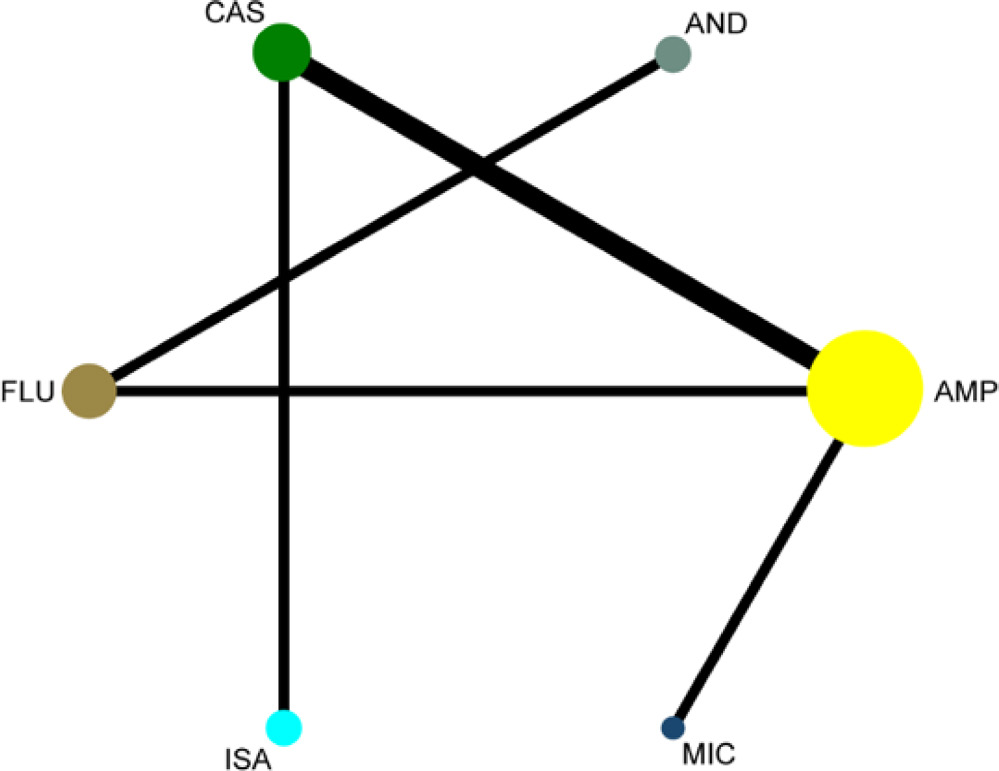
Network graph of included studies reporting outcomes. Each node represents a therapy with the thickness of the line and size of the circle proportional to the number of studies and number of participants, respectively, in the head-to-head comparison. Abbreviations: CAS, Caspofungin; AND, Anidulafungin; AMP, Amphotericin B; MIC, Micafungin; ISA, Isavuconazole; FLU, Fluconazole.

**Fig. 4.**
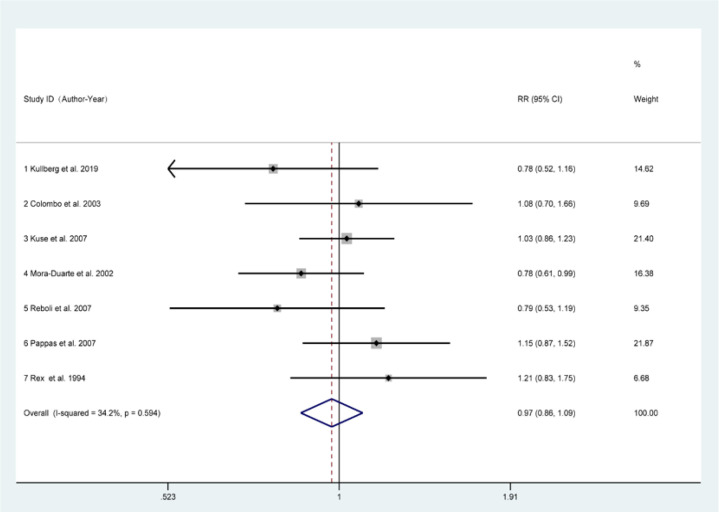
Forest plot of included studies for comparing the overall efficacy of antifungal drugs in the included studies.

Using a pair-wise meta-analysis to compare the overall efficacy of antifungal drugs, the sizes of the nodes reflect the numbers of participants, and the widths of the lines indicate the numbers of included trials. The results showed a significant benefit in favour of antifungal inventions for *C. parapsilosis* infections (Fig. 3). We next performed a meta-analysis comparing primary outcomes achieved with various antifungal agents. The forest plot results showed that antifungal treatment promoted good outcomes with an overall pooled relative risk (RR) of 0.97 (95 % CI :0.86–1.09). Based on the chi-square and I^2^ analyses, small differences in heterogeneity were observed between the treatment groups [I^2^=34.2 %] (Fig. 4).

### Network meta-analysis of antifungal treatment

**Fig. 5.**
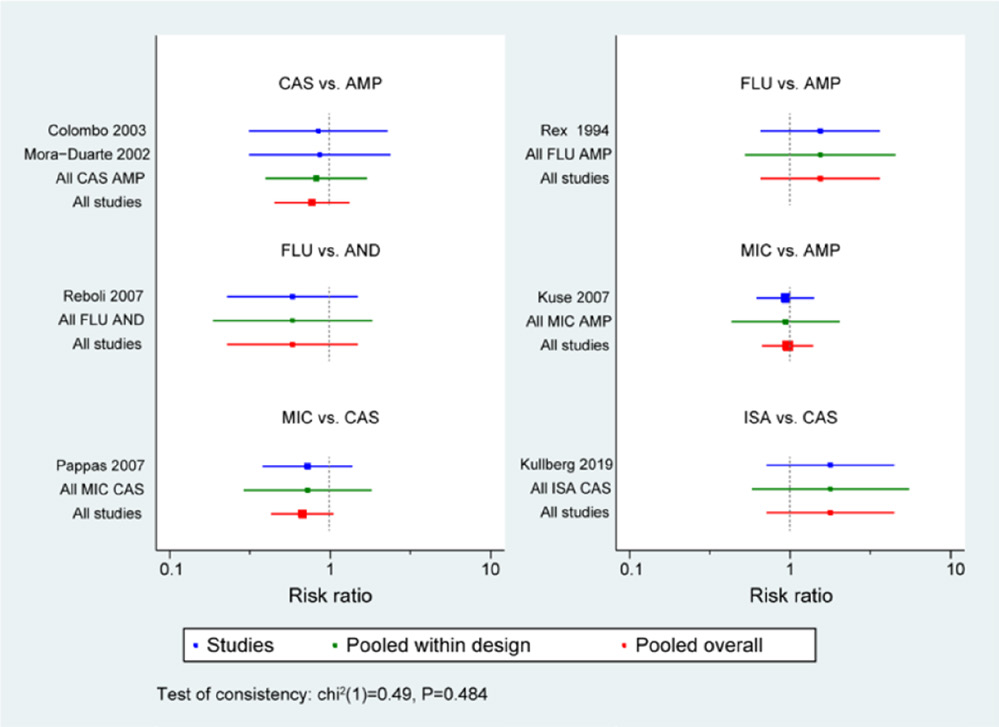
Evidence-network analysis for paired-comparison of the efficacy of antifungal drugs in the included studies.

**Fig. 6.**
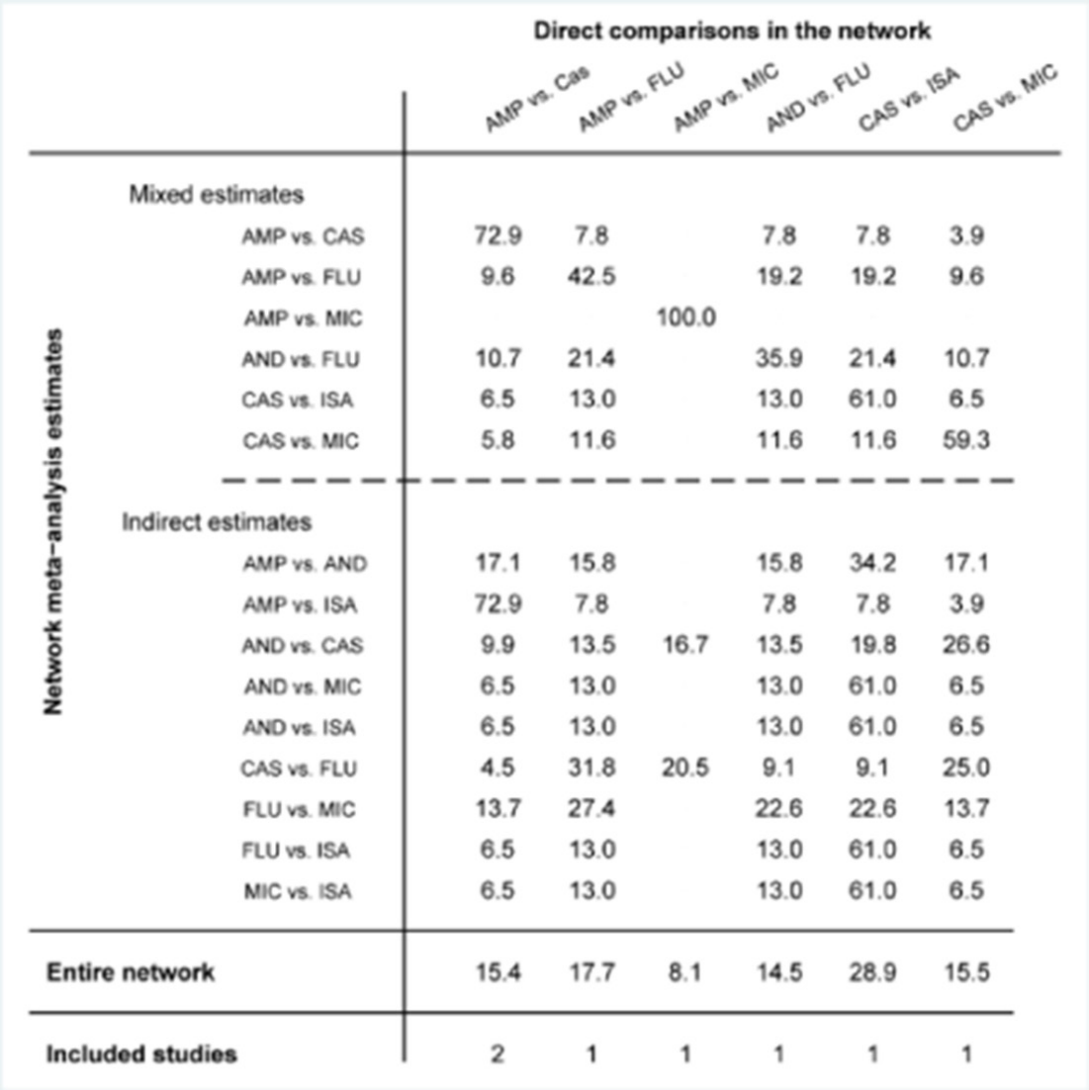
Contribution plot for the included studies.

To compare the different effects of antifungal treatments for *C. parapsilosis* infections**,** we then used the network meta-analysis to explore whether there were differences among different antifungal drugs in patients with *C. parapsilosis* infections. First, we performed an evidence-network analysis for different treatments and generated a forest plot for every included study with direct and indirect analysis (Fig. 5). The results demonstrated that CAS presented a better efficacy than AMP or ISA against *C. parapsilosis* infections. Furthermore, all of the drugs, including FLU, MIC, ISA, AMP, CAS and AND were compared separately with each other, and Fig. 6 shows the contribution plot for the included publications in the network. The most informative direct evidence in the network was CAS vs. ISA with an overall contribution of 28.9 % to the network estimates.

**Fig. 7.**
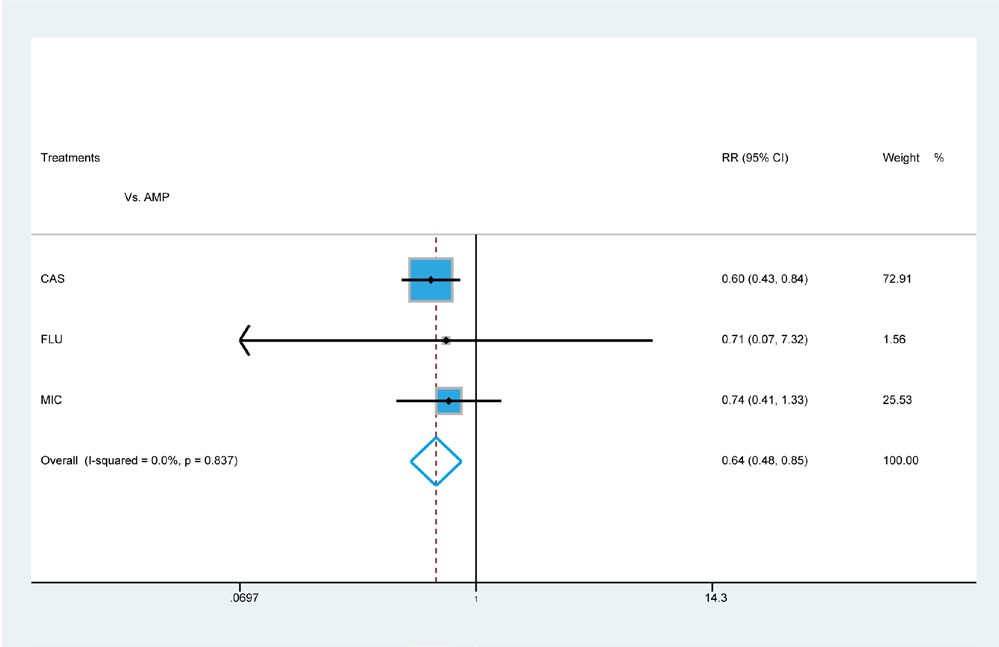
Comparisons of 30 day mortality. Summarized RR and corresponding 95 % CI for 30 day mortality comparing multiple treatments.

To explore the risks of death with antifungal agents, we analysed the included studies reporting 30 day mortality for patients with *C. parapsilosis* infection. In the subgroup analysis, compared with patients who received AMP, patients treated with CAS (RR 0.60, 95 % CI 0.43–0.84) presented with a significantly lower risk of 30 day mortality than those who received other treatments (Fig. 7).

### Consistency and publication bias assessment

**Fig. 8.**
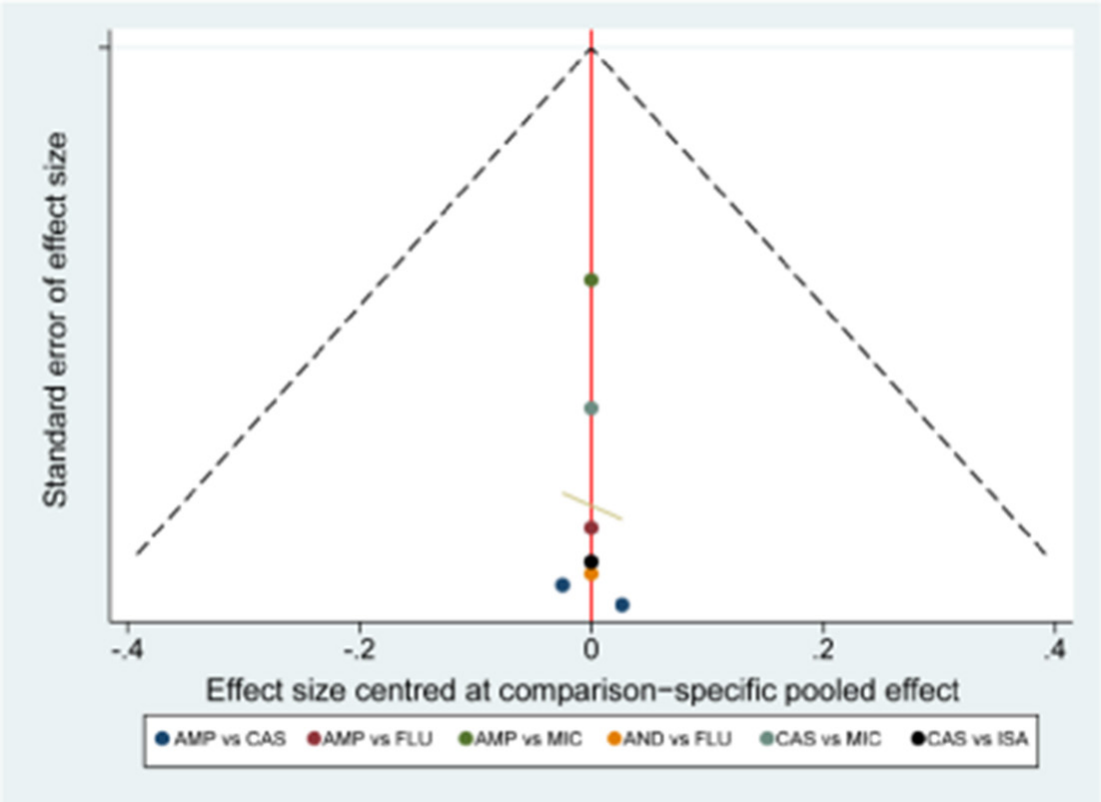
Comparison-adjusted funnel plot for the network meta-analysis.

To assess the inconsistency among the included studies, node-splitting models were applied by testing the difference between the direct and indirect comparisons (Fig. 8). The results of the consistency model were reliable, which demonstrated good convergence and efficiency. The comparison-adjusted funnel plot for the efficacy of these seven treatments showed that there was no publication bias based on Begg’s test (*P*=0.61) among the included studies.

## Discussion

Systemic fungal infections caused by conditioned pathogens such as non-*albicans Candida* including *C. parapsilosis* have emerged along with a gradual increase in blood-stream infections in healthcare settings with the widespread application of broad-spectrum antibiotics, immunosuppressants, and anti-malignant drugs, increased performance of organ transplantation, advances in medical support technology, the extension of human life, as well as the increase in the prevalence of acquired immune deficiency syndrome (AIDS) [22–24]. Research on the pathogen *C. parapsilosis* and exploration of its pathogenic mechanism are underway [25].

Antifungal drugs are currently the most effective method for the treatment of *Candida* infections [26, 27]. AMP serves as a representative of polyene antifungal drugs and has been widely used in the treatment of fungal infections, especially severe fungal infections [28]. It has been reported that AMP is more than 70 % effective in treating fungal infections. However, it has several obvious side effects, especially nephrotoxicity. The first-generation azoles such as FLU, itraconazole, and voriconazole show relatively good efficacy for reducing the death rate among haematological stem cell transplant recipients [29]. However, the bioavailability of itraconazole varies greatly, and drug resistance to FLU develops readily [30]. In contrast, the newer triazoles such as voriconazole and posaconazole show a broader antibacterial spectrum, higher bioavailability, and significantly fewer adverse reactions than the first-generation triazole drugs [31]. Echinocandins such as micafungin target 1,3-β-D glucan synthase, inhibit the synthesis of glucan synthase, and interrupt formation of the cell wall, ultimately leading to cell death [32]. Caspofungin was the first echinocandin drug approved by the US Food and Drug Administration (FDA) and proven to be comparatively safe and efficacious against *Candida* species, including *C. albicans*, and non-*C. albicans* [33].

To determine the ideal antifungal drugs for the treatment of *Candida* infections, many studies have compared the clinical outcomes of different antifungal drugs. The outcomes of different therapies in whole *Candida* infections have been widely reported; however, reports specifically on *Candida parapsilosis* infections are rare, especially with direct comparison. If differences in outcome were observed among those different therapies, the related studies are very limited and only several studies have reported a difference in treatment outcomes. In the present study, a network meta-analysis was performed including seven RCTs with a total of 2434 patients, and most studies were compared different clinical outcomes. Therefore, we analysed the clinical efficacy of treatments for *C. parapsilosis* infections. The main results of our study showed the efficacy of antifungal inventions. Among different target outcomes, with the evidence-network analysis, the efficacy of CAS was better than that of other drugs for treating *C. parapsilosis* infections from both the direct and indirect analyses of the included studies. These findings were slightly inconsistent with those of a previous prospective cohort study exploring the effectiveness of echinocandins including micafungin, caspofungin, and anidulafungin as empirical antifungal drugs for the treatment of invasive *Candida* infections, which found no significant difference. However, in the present study focused on the treatment efficacy of antifungal drugs, we revealed that the efficacy of antifungal treatment in the echinocandin group was significantly lower than that in the other groups.

CAS is an echinocandin that inhibits fungal growth by the inhibition of 1,3-β-glucan synthase and preventing fungal cell wall synthesis. CAS is being increasingly used as first-line therapy for invasive candidiasis. Clinical studies and *in vitro* susceptibility data have indicated that CAS is at least as active as AMB and FLU in the treatment of invasive candidiasis, and according to empirical therapy, caspofungin was superior to AMP and voriconazole for the outcome of survival [34]. Additionally, previous studies demonstrated that CAS is effective against *C. parapsilosis* and active in experimental systemic candidiasis caused by *C. parapsilosis*. More importantly, our study further confirmed that CAS showed a preferable effect on decreasing the risk of 30 day mortality.

This study has certain limitations. First, there is still a lack of similar high-quality clinical trials, and the number of included studies and the sample sizes were small. Second, the criteria for determining the effectiveness outcomes could not determine whether they were uniform. Third, the sample size for CAS treatment in the included studies was much larger than those for other treatments; therefore, the results may lead to a conclusion in favour of CAS treatment. Fourthly, the included studies did not cover all primary and secondary outcomes or the dosage of antifungal drugs. Finally, there may be sampling bias from using studies published before June, 2020 and new trials relevant to this topic may be published or updated later. The meta-analysis could be updated in the future with enough time to check for enough new evidence.

In summary, this study systematically evaluated the effectiveness and safety of antifungal drugs, analysed and summarized the status and problems of fungal drugs in clinical treatment, and aimed to help clinicians choose the most appropriate antifungal drugs and improve the level of clinical treatment. Additionally, the study provides useful data for the development of standardized dosing regimens and standardized diagnostic criteria, and high-quality, multi-centred and real-word studies with a long-term follow-up are needed to further explore and verify the efficacy of antifungal drugs for the prevention and treatment of *C. parapsilosis* infection.

### Availability of data and material

The datasets generated and analysed in the present study are available from the corresponding author upon reasonable request.
